# Sequence level genome-wide associations for bull production and fertility traits in tropically adapted bulls

**DOI:** 10.1186/s12864-023-09475-2

**Published:** 2023-06-29

**Authors:** Wei Liang Andre Tan, Laercio Ribeiro Porto Neto, Antonio Reverter, Michael McGowan, Marina Rufino Salinas Fortes

**Affiliations:** 1 School of Chemistry and Molecular Biosciences, The University of Queensland, Chemistry Bld, 68 Cooper Rd, Brisbane City, QLD 4072 Australia; 2 CSIRO Agriculture and Food, 306 Carmody Road, St Lucia, QLD 4067 Australia; 3 School of Veterinary Science, The University of Queensland, Gatton, QLD 4343 Australia

**Keywords:** Genome-wide Association study, Bull fertility, Quantitative trait loci, Sequence-level

## Abstract

**Background:**

The genetics of male fertility is complex and not fully understood. Male subfertility can adversely affect the economics of livestock production. For example, inadvertently mating bulls with poor fertility can result in reduced annual liveweight production and suboptimal husbandry management. Fertility traits, such as scrotal circumference and semen quality are commonly used to select bulls before mating and can be targeted in genomic studies. In this study, we conducted genome-wide association analyses using sequence-level data targeting seven bull production and fertility traits measured in a multi-breed population of 6,422 tropically adapted bulls. The beef bull production and fertility traits included body weight (Weight), body condition score (CS), scrotal circumference (SC), sheath score (Sheath), percentage of normal spermatozoa (PNS), percentage of spermatozoa with mid-piece abnormalities (MP) and percentage of spermatozoa with proximal droplets (PD).

**Results:**

After quality control, 13,398,171 polymorphisms were tested for their associations with each trait in a mixed-model approach, fitting a multi-breed genomic relationship matrix. A Bonferroni genome-wide significance threshold of 5 × 10^− 8^ was imposed. This effort led to identifying genetic variants and candidate genes underpinning bull fertility and production traits. Genetic variants in *Bos taurus* autosome (BTA) 5 were associated with SC, Sheath, PNS, PD and MP. Whereas chromosome X was significant for SC, PNS, and PD. The traits we studied are highly polygenic and had significant results across the genome (BTA 1, 2, 4, 6, 7, 8, 11, 12, 14, 16, 18, 19, 23, 28, and 29). We also highlighted potential high-impact variants and candidate genes associated with Scrotal Circumference (SC) and Sheath Score (Sheath), which warrants further investigation in future studies.

**Conclusion:**

The work presented here is a step closer to identifying molecular mechanisms that underpin bull fertility and production. Our work also emphasises the importance of including the X chromosome in genomic analyses. Future research aims to investigate potential causative variants and genes in downstream analyses.

**Supplementary Information:**

The online version contains supplementary material available at 10.1186/s12864-023-09475-2.

## Background

Northern Australia represents a critical region for the Australian beef breeding industry, and bull fertility is an important contributor to profitability [1–3]. However, bull fertility has yet to benefit from the advancements in genomics and selective breeding, which has further contributed to improving female fertility [4]. The Bull Breeding Soundness Evaluation (BBSE) provides a comprehensive assessment of male fertility-related traits linked to the number of calves a sire produces in the subsequent mating season [5, 6]. The BBSE traits which consist of assessment of body conformation, testicular development and sperm motility and morphology assessment, are heritable and can be used for selection and genetic improvement programs [7]. Previous genome-wide association studies (GWAS) have identified candidate genes for Scrotal Circumference (SC) and semen traits which are recorded in BBSE [8–11]. Identifying these critical genomic regions expands the current understanding of the underlying genetics of bull fertility and can also be used to inform genomic predictions and improve their accuracy [12].

Previous work used medium or high-density SNP arrays, such as the Illumina 50 K panel or the BovineHD chip. Thus, genetic variants associated with GWAS are usually not causal mutations but are single nucleotide polymorphisms (SNP) in Linkage Disequilibrium (LD) to causal variants [13]. With advancements in genome sequencing and imputation methodologies, lower-density panels can be accurately imputed to sequence level [14, 15]. This allows the genome to be viewed in finer detail, which increases our chances of detecting a causal variant. This study aimed to conduct GWAS on seven BBSE traits to identify genetic variants and candidate genes underpinning bull fertility. The variants identified in this analysis could be incorporated into genomic predictions to improve the rate of genetic improvement in bull fertility and production traits.

## Materials and methods

### Animals and phenotypes

A total of 6,422 animals of six breeds with BBSE measurements of seven phenotypes were used in this study. These animals are from two research populations and four stud herds from the industry. The two-research populations consisted of animal data obtained from the Cooperative Research Centre for Beef Genetic Technologies (Beef CRC) project [16], which included 1,051 Brahman (BRH) and 1,819 Tropical Composite bulls (TRC). Animal data for these four stud herds were contributed by four properties in Queensland, which included 1,288 Santa Gertrudis (SGT), 760 Droughtmasters (DMT), 844 Ultra blacks (UBK), and 660 Belmont Tropical Composite (BTC) [17]. The seven BBSE phenotypes used in this study included four physical measures on the animal and three semen measurements. These measurements were conducted according to the standards prescribed by Australian Cattle Veterinarians [5], which have been covered extensively in the literature [16]. Details on the seven phenotypes can be found in Table 1. Summary statistics and heritabilities of each trait are shown in Table 2. The breed-wise summary statistics for each trait are available in Additional file 6.

**Table 1:** Traits measured as part of Bull Breeding Soundness Evaluation (BBSE).

Traits^A^	Description^B^
Body conformation	
Weight	Body weight (Kg)
Body condition score (CS)	Visual assessment of body condition: Scale 1–5.
Sheath score (Sheath)	Visual score of the structure of the sheath. Scale 1(Pendulous) to 5(Very tight).
Scrotal circumference (SC)	Scrotal circumference measured at the largest circumference of the scrotum (cm).
Laboratory semen assessments	
Percentage of Normal Sperm (PNS)	Microscopic analysis. The percentage of anatomical normal sperm in a given sample. Assessment based on standards for shape indicating normal or abnormal cells.
Percentages of specific abnormalities	Microscopic analysis. Percentage of specific sperm defects (abnormalities). Percentage of sperm having proximal cytoplasmic droplets (PD), or mid-piece abnormalities (MP).

^A^Phenotype column heading information, ^B^Description of phenotype and scoring metric

**Table 2:** Summary statistics and summary of GWAS results

	N^A^	Mean^B^	SD^C^	Min^D^	Max^E^	Adj Min^F^	Adj Max^G^	SNPs^H^	h^2^(SD)^I^
Weight	6014	391.59	98.65	124.00	810.00	-175.60	268.42	169	0.30 (0.02)
CS	5917	2.96	0.37	2.00	4.00	-1.52	1.13	30	0.07 (0.02)
SC	6235	30.82	4.26	15.50	52.50	-9.59	15.96	55,706	0.46 (0.02)
Sheath	6417	3.19	1.77	1.00	9.00	-3.97	6.78	135,424	0.59 (0.02)
PNS	6055	61.76	27.53	0.00	100.00	-76.44	65.61	9042	0.18 (0.02)
PD	6052	13.50	19.96	0.00	96.00	-48.41	84.78	1726	0.15 (0.02)
MP	6052	11.39	11.04	0.00	83.00	-22.94	68.73	173	0.13 (0.02)

^A^Number of records available for a trait. ^B^Mean of a trait. ^C^Standard deviation of a trait. ^D^Minimum value of the trait. ^E^Maximum value of the trait. ^F^Minimum adjusted value of the trait. ^G^Maximum adjusted value of the trait. ^H^Number of significant SNPs identified using LOCO GWAS analysis. ^I^Heritability estimates for each trait

Phenotypic measures for all six populations were collected from 2003 to 2020. Each bull was assessed once, and the year of measurement was recorded as the fixed effect - year of birth. The individuals involved in the assessment and collection of phenotypes are different for the two research populations and the four stud herds. Phenotypes for the two research populations were collected and assessed by two experienced veterinarians who worked together throughout the collection period. In the four stud herds, an experienced animal scientist and veterinarian conducted the examinations. For semen morphology traits, semen samples from the two research populations were analysed in the same laboratory. Whereas, sperm samples from the four stud herds were assessed in different accredited labs selected by the producers. The age of the bulls when the phenotypes were collected differed across the six populations. In the Beef CRC, when SC was collected, BRH and TRC bulls were around 360 days old. Sheath score and sperm morphology assessments were conducted at around 700 days old. In the four stud herds, all phenotypes for SGT and DMT bulls were obtained at around 600 days, whereas UBK and BTC bulls had their phenotypes measured around 440 days old and 390 days old, respectively. Phenotypes were pre-adjusted using a generalised linear model analysis (PROC GLM) for their fixed effects (year of birth, breed, property) and covariates (age at measurement, PC1 and PC2) using SAS ® software 9.4 (SAS Inst. Inc.). A subset of this population was previously used in a multi-breed analysis [18].

### Genotypes, quality control and genomic relationship matrices

SNP genotype imputation up to whole-genome sequence level was conducted in two rounds. In the first round, the reference population was established using Beef CRC and industry cattle. This reference population consisted of 2,452 animals made of BTC, BRH, DMT, SGT, UBK, Angus, Bonsmara, Boran, Composite, and Tuli breeds that were genotyped with the bovine high-density chip (~ 700 K) and phased using Eagle 2 (v2.4.1) which formed the imputation targets for the next step [19]. In the target population (n = 6422), genotyping was first done using a variety of commercial 50 K SNP chips (Bovine SNP50 v1 or v2 or Neogen Tropical Chip v1 and v2). The genotypes from these animals were also phased with Eagle 2 (v2.4.1) [19], and formed the imputation targets for the next step. Imputation of targets from low to high density using the phased reference was conducted using Minimac3 for the autosomes and Minimac4 for the X chromosome [14]. For imputation to sequence level, genotypes from 668 animals in the 1000 bull genome project run 7 [20] were filtered to keep only bi-allelic markers and minor alleles with at least four copies. The sequence-level reference panel consisted of 668 animals made of BRH, DMT, SGT, Afrikander, Angus, Angus Red, Beefmaster, Boran, Brangus, Charolais, Gir, Hereford, Limousin, Murray Grey, Nelore, Senepol, Shaiwal, Shorthorn and Tuli breeds. The data generated in the first round was then used to impute genotypes to sequence level (~ 25 million) in the second round using the same procedure done in the first round. SNPs with an imputation R^2^ > 0.8, a call rate > 0.9 and a minor allele frequency > 0.01 were kept for further analysis, leaving 13,398,171 SNPs, including 92,134 SNPs mapped onto the X chromosome after quality control for all 6422 animals. The Genomic Relationship Matrices (GRM) were constructed in the software GCTA [21] using a high-density panel, one GRM for the autosomes plus the Pseudo – Autosomal Region of chromosome X (657,563 SNPs for autosomes and 22,775 SNPs for X), and a second GRM using the remaining SNP from the X chromosome. Heritability estimates for each trait were obtained using the restricted maximum likelihood (REML) analysis in GCTA [21].

### Genome-wide association analysis and quantitative trait loci analysis

The first two principal components calculated PLINK 1.9 [22], and the GRMs, were used to account for the underlying genetic structure of the multi-breed population under study. As bias could be introduced when a tested SNP is also included in the GRM that is fitted in the model [23], the Leave One Chromosome Out (LOCO) approach to GWAS was also implemented by building a different GRM when testing each chromosome, leaving out any SNPs that are on the tested chromosome [24]. The MLM method implemented in GCTA is as follows:

$$y=a+bx+{g}^{-}+e$$

Where y represents the phenotype in question, a represents the mean, b represents the additive genetic effect of the tested SNP, x represents the SNP genotype indicator variable which is coded as 0, 1 and 2, g ^–^ represents the joined effect of all variants, excluding any variants on which the chromosome of the tested SNP is located, and e is the residual variance. A genome-wide significance threshold of 5 × 10^− 8^ was used, which is a conservative Bonferroni correction. After the first round of GWAS was completed, the most significant SNPs in each chromosome were refitted as a discrete covariate in the second round of GWAS for each trait in GCTA [21]. This was done to determine if the most significant SNP in each chromosome could account for the entire peak for that chromosome. GWAS Manhattan plots were created in R [25] using the Scattermore package [26]. Using bedtools [27], SNPs within 50 Kbp that met the significance threshold (5 × 10^− 8^) were merged into a Quantitative Trait Loci (QTL). Using GALLO [28], genes found in each region were reported using gene annotation data of the *Bos taurus* ARS UCD 1.2 genome assembly obtained from Ensembl version 105 [29]. The find_genes_qtls_around_markers function was used to identify the genes located in each region. The following parameters were used: the method was set to gene, marker was set to haplotype, and the interval was set to 0.

Similarly, the same regions were used to identify any previously reported QTL in the Animal QTL database (https://www.animalgenome.org/cgi-bin/QTLdb/BT/index) [30] that overlapped with regions reported in this study. Ensembl Variant effect prediction (VEP) was conducted on all significant SNPs to ascertain the impact of each variant [31]. Pairwise LD was calculated between the high impact variants and the top variant for their respective QTL using PLINK 1.9 [22]. We considered variants that meet the R^2^ threshold of 0.4 to be in LD. Finally, the percentage of genetic variance explained by each SNP was calculated using a formula made available in a previous report [32]:

$$\%{V}_{i}= 100 x \frac{2{p}_{i}{q}_{i}\widehat{{a}_{i}^{2}}}{{\sigma }_{g}^{2}}$$

Where p_i_ and q_i_ are the SNP’s allele frequencies $\widehat{{a}_{i}^{2}}$ is the estimated additive effect of the trait studied, and ${\sigma }_{g}^{2}$ is the estimated genetic variance.

## Results and discussion

**Fig. 1:**
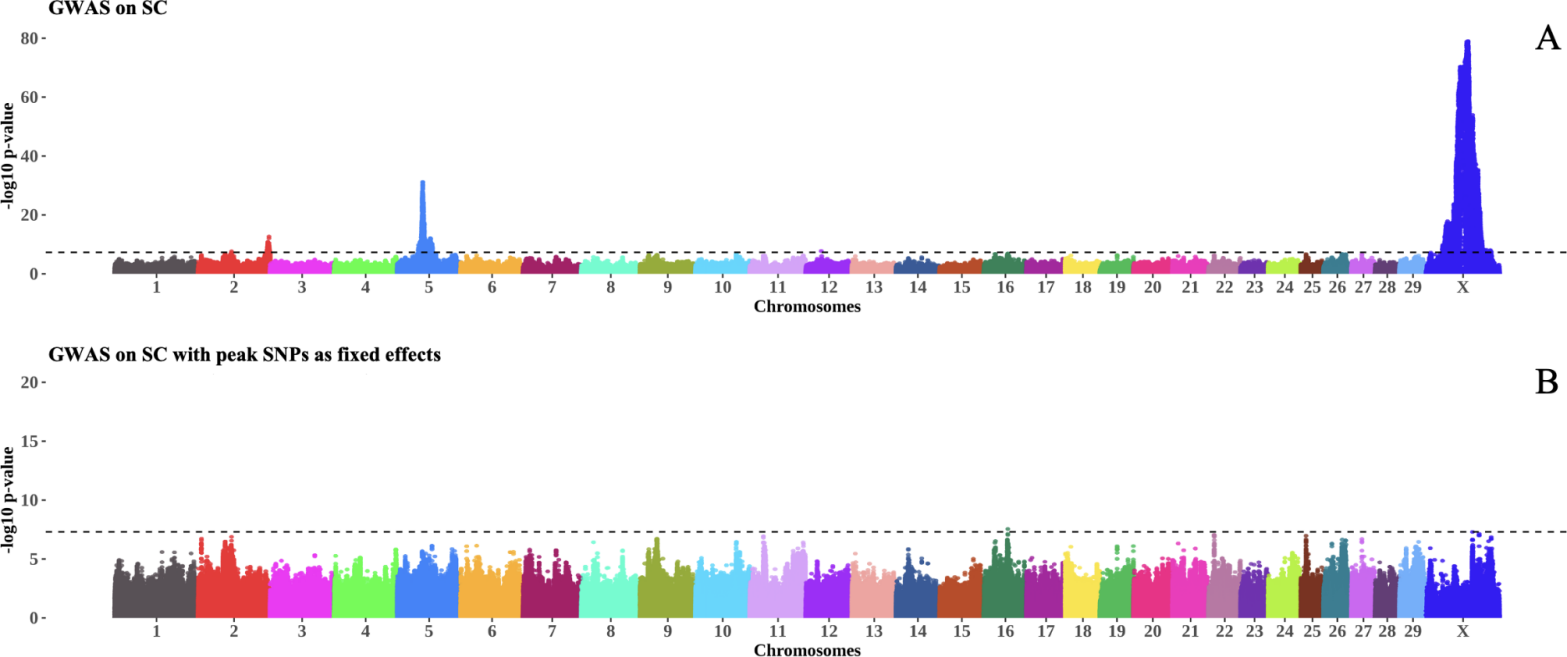
The Manhattan plot in **A** shows associations for SC in GWAS LOCO, whereas the Manhattan plot in **B** shows associations for SC after fitting the most significant SNP in each chromosome as a fixed effect. The inverse log p – values for each SNP are plotted along the y-axis for each chromosome on the x-axis. The dotted line represents the genome-wide significance threshold of 5 × 10^− 8^

**Fig. 2:**
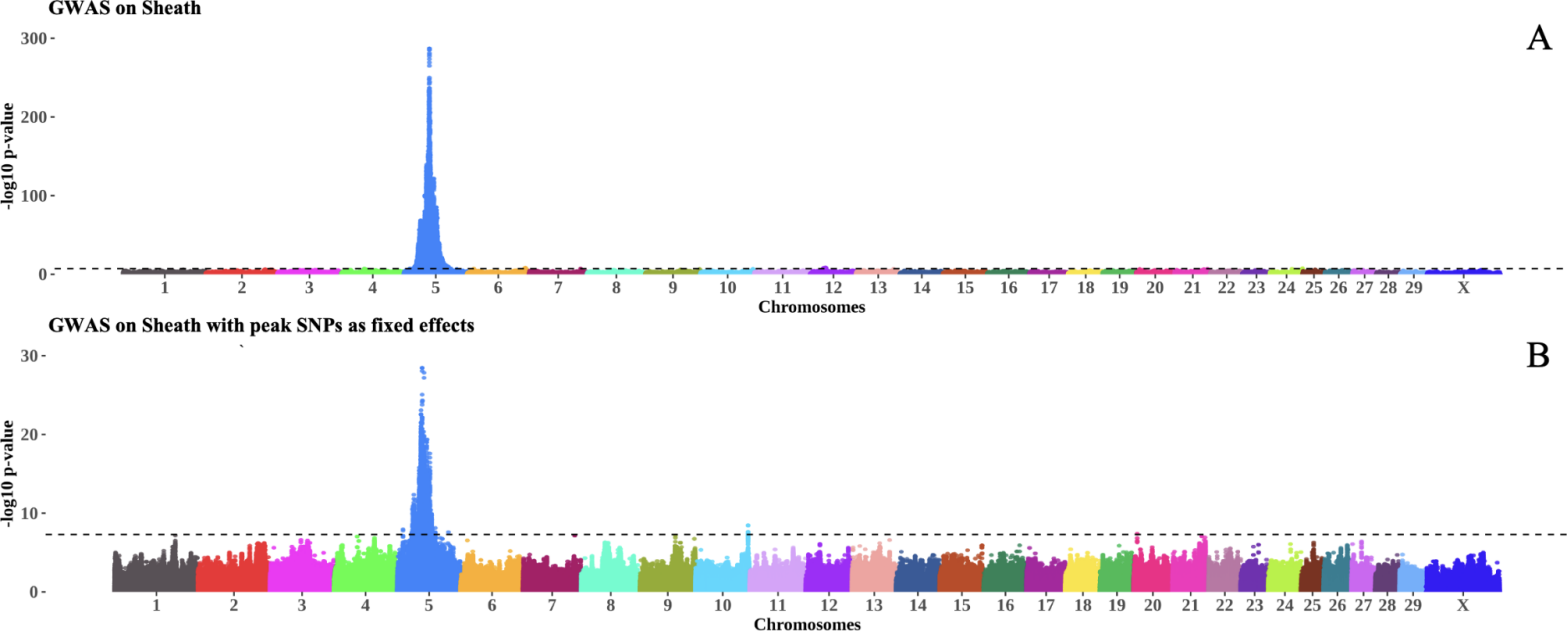
The Manhattan plot in **A** shows associations for Sheath in GWAS LOCO, whereas the Manhattan plot in **B** shows associations for Sheath after fitting the most significant SNP in each chromosome as fixed effects. The inverse log p – values for each SNP are plotted along the y-axis for each chromosome on the x-axis. The dotted line represents the genome-wide significance threshold of 5 × 10^− 8^

**Fig. 3:**
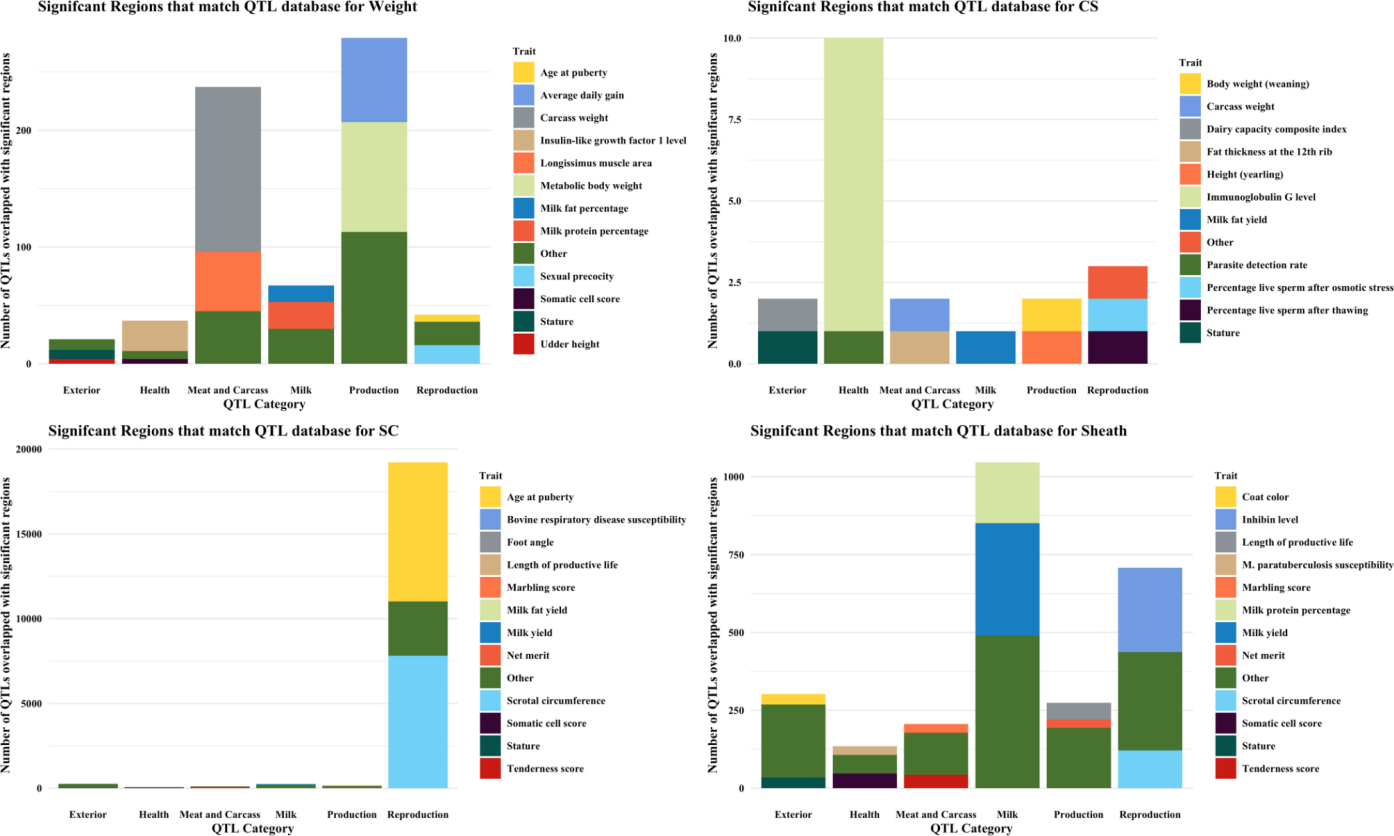
Stacked bar plots showing published QTL and their associated traits that overlapped with QTL reported in this study for body conformation traits

**Fig. 4:**
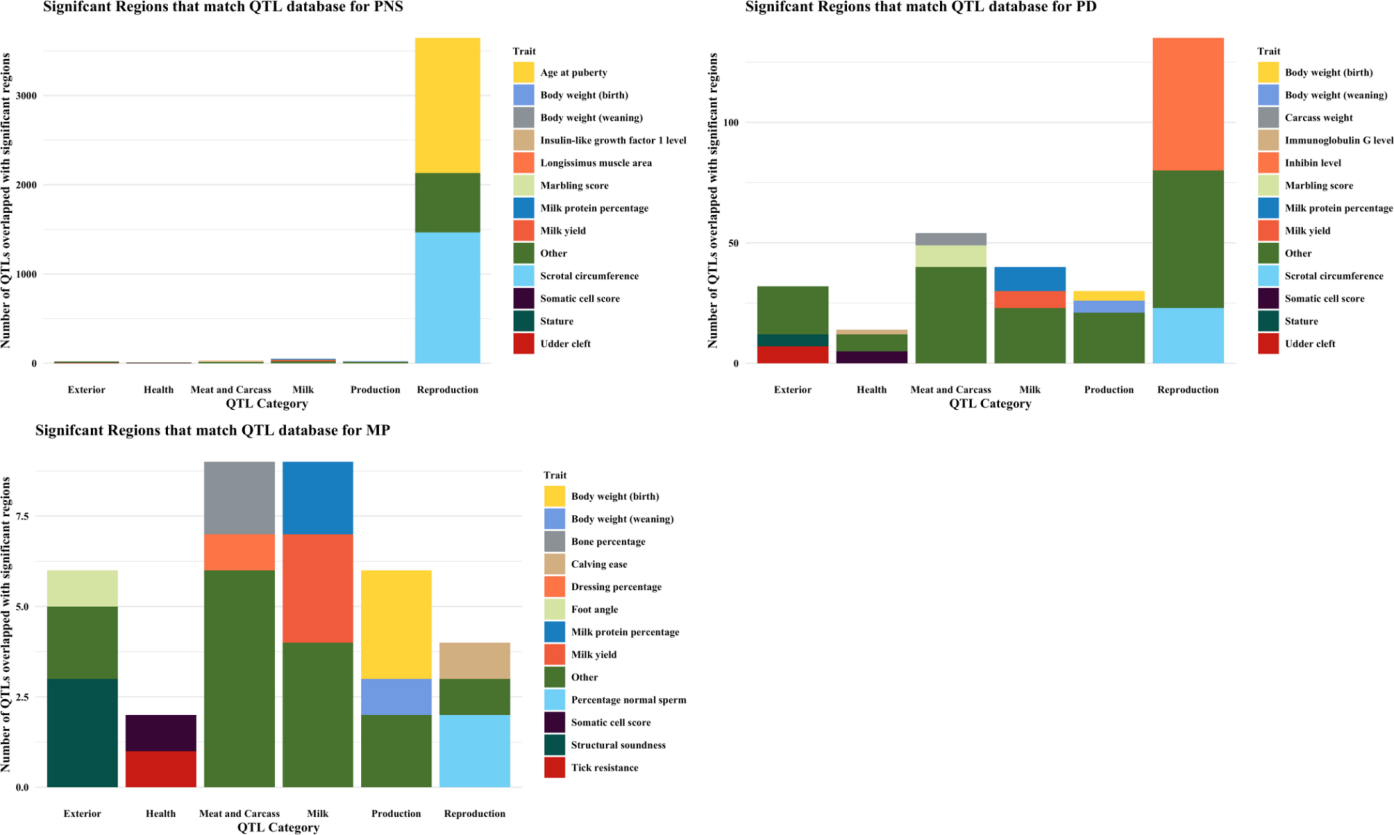
Stacked bar plots showing published QTL and their associated traits that overlapped with QTL reported in this study for semen traits

In this study, we conducted GWAS using sequence-level genotypes and targeted seven bull fertility and production traits measured in a multi-breed population of 6,422 bulls. This section discusses important regions and candidate genes identified through GWAS and QTL analysis. A summary of GWAS results for the most significant genomic region discovered for each trait is provided in Table 3. A complete table of GWAS summary statistics for all tested SNP and each trait is available in Additional file 1. Additional file 2 contains SNP that were significant for at least one trait. Manhattan plots for GWAS in SC and Sheath are shown in Figs. 1 and 2. The remaining Manhattan plots can be found in Additional file 3. A vast number of previously published QTL were identified for some traits. As such, we have summarised these results in Figs. 3 and 4. The sperm morphology traits (PNS, PD, and MP) did not have normally distributed residuals. This is not ideal for GWAS, but it is expected as sperm morphological abnormalities affect only some bulls. The majority of breeding bulls present a high percentage of normal sperm.

**Table 3:** Summary of the most significant genomic region per trait

Trait^A^	Main Peak			N SNP < 5 × 10^− 8 E^	Most significant SNP^F^	Gene and consequence^G^
	**Chr** ^B^	**Start-End** ^C^	**P-Value** ^D^			
Sheath	5	44,264,399–57,689,597	8.44 × 10 ^− 288^	46,571	5:47810529 rs132782818	LLPH, Downstream Gene Variant
PNS	5	46,332,642–46,628,009	3.35 × 10^− 14^	436	5:46434577 rs385064678	CAND1, Intergenic Variant
PD	5	45,878,759–46,630,417	1.98 × 10^− 13^	739	5:46035763 rs717912623	DYRK2, Intergenic
WT	14	23,278,231–23,392,939	2.27 × 10^− 10^	25	14:23338890 rs109815800	PLAG1, Intron Variant
CS	23	6,692,720–6,752,357	1.79 × 10^− 9^	10	23:6704516 rs720629044	LRRC1, Intron Variant
SC	X	78,997,703–82,589,295	1.15 × 10^− 79^	2986	X:79,072,719 rs132656042	CXCR3, Downstream Gene Variant
MP	X	6,803,110–6,845,284	2.77 × 10 ^− 10^	24	X:6,803,111 rs479067101	GRIA3, Intergenic Variant

^A^Corresponding trait. ^B^Chromosome number of the most significant region genome-wide ^C^Start and end location of the most significant region genome-wide. ^D^P-value of the most significant variant Genome-wide. ^E^Number of SNPs that meet the significance threshold within the most significant region. ^F^Location of most significant SNPs and Reference SNP cluster ID. ^G^Nearest gene and predict consequence from Ensembl Variant Effect Predictor

### Heritability estimates of individual traits

Heritability estimates across traits range from low (0.07, CS) to high (0.59, Sheath) (Table 2). The estimates we report for our traits were similar to those published in tropical beef cattle populations [8, 33, 34]. Our estimates for SC were similar to those measured in TRC bulls at 12 months (0.46) and slightly higher than those measured at 24 months (0.44) [34].

#### Single trait associations

The number of associated SNP varied enormously, depending on the target trait (Table 3). A total of 30 significant SNPs were detected for CS, while more than 135 thousand SNP were significant for Sheath. The strongest SNP association for Weight was located at 23.3 Mb of BTA 14 (*p* = 2.27 × 10^− 10^). This result is somewhat expected because previous GWAS in datasets containing *Bos Taurus* and *Bos Indicus* breeds have been reporting a QTL in BTA 14 for stature and weight traits [35–37].

The strongest SNP association for CS (*p =* 1.79 × 10 ^− 9^) was located at 6.7 Mb of BTA 23. This is a new discovery as there are no CS QTL in BTA 23 currently recorded in the cattle QTL database (https://www.animalgenome.org/cgi-bin/QTLdb). The strongest SNP association for SC (*p* = 1.15 x ^− 79^) was located at 79 Mb on the X chromosome. This is not the first time we detect SNP associations on X for SC, and so this result confirms previous GWAS carried out with smaller datasets [8, 9, 11]. The strongest association (*p* = 1.98 × 10^− 288^) for Sheath was located at 47.8 Mb of BTA 5. This finding is consistent with previous GWAS work in sheath score that used a subset of the data included in this study [32]. The subset of data contained only BRH and TRC bulls, which differs from our multi-breed analyses [32]. In short, the larger dataset is expanding on the initial findings and in subsequent sections of this discussion, we detail the QTL, genes, and variants uncovered with sequence-level data.

Similarly, the current dataset enhanced our ability to detect associations for the three semen traits: PNS, PD and MP. The strongest SNP association (*p* = 3.35 × 10 ^− 14^) for PNS was located at 46.4 Mb at BTA 5. Previously, we had only identified SNPs on X for PNS [8, 9, 11]. A recent study on American cattle did identify SNP associations in chromosome 5 for PNS, corroborating our new finding in tropical breeds [38]. The strongest SNP association (p = 1.98 × 10 ^− 13^) for PD was located at 46 Mb of BTA 5. A total of 173 significant SNP associations were detected for MP. The strongest SNP association (*p* = 2.77 × 10 ^− 10^) was located at 6.2 Mb of the X chromosome. This multibreed dataset confirmed that chromosome X harbors SNP associations for semen traits as expected [8, 9, 11]. It also allowed the discovery of significant SNP on BTA 5, pointing to new candidate genes (described below).

The most significant SNP for a trait may not account for all the variation at a particular locus, and multiple causal variants may exist at a given locus [39]. As such, we verified the most significant SNPs for each chromosome in each trait by refitting these SNPs back into the mixed model. In general, the most significant SNP in each chromosome accounted for the entire variation in that locus for most traits, as seen in Figs. 1 and 2. However, in Sheath, the most significant SNP did not account for all the variation in BTA 5 (Fig. 2). Perhaps more than one causal SNP exists in that BTA 5 region and this is important because it overlaps with significant QTL discovered for SC and semen traits. The SNP associations across traits found in BTA 5 are discussed in more detail below (see Table 4).

**Table 4:** Summary of the number of QTL and the sum of all QTL for each trait

Trait^A^	Number of QTL^B^	Total sum of QTL (bp)^C^
Sheath	85	50,798,093
PNS	69	13,312,033
PD	42	1,885,633
WT	22	722,476
CS	3	107,850
SC	148	72,863,848
MP	15	435,628

^A^Corresponding Trait. ^B^Number of significant QTL. ^C^Total sum of QTL in base-pair

### QTL analysis

The GWAS literature includes accounts of false positives: QTL or SNP associations that are seen once and not validated (winners curse) [40]. To mitigate this issue, we focused on reporting QTL regions that overlap with known QTL from previous work. We used the QTL database [30] to identify consensus between our current analyses and published work. The number of significant QTL identified per trait and the total sum of these QTL can be found in Table 4.

A total of 1,120 previously reported QTL overlapped with the regions identified in this study for weight. While most of the identified QTL were associated with weight-related traits, some of these regions were also important female traits such as milk fat yield, health-related traits, and reproductive traits.

A total of 20,095 previously reported QTL overlapped with the significant regions reported for SC. Most QTL were associated with SC reported in Canchim bulls [41], and the age of puberty was reported in our previous study with TC bulls [11]. This result is not surprising as a bull is considered to have reached puberty after achieving a SC of 26 cm [42].

In Sheath, 2,671 previously reported QTL overlapped with the significant regions in this study. Most QTL were associated to female traits such as milk protein percentage or milk yield. However, QTL were also associated to male traits such as inhibin hormone levels and SC. Inhibin hormone levels are considered an early indicator of sexual development, and genes such as INHBE and INHBC are located in BTA 5 [11, 43]. Of note, the GWAS on blood hormone levels of Inhibin used 50 K genotype data for Brahman and TRC cohorts that were included in the current larger dataset [11].

Previously reported QTL for PNS mirrored the result for SC (Figs. 3 and 4). This is not surprising given that both BTA 5 and chromosome X were associated with both traits, and a positive genetic correlation has been reported in a previous study between the two traits [16]. For PD, previously reported QTL were associated with reproduction traits such as inhibin level and SC, whereas most QTL were associated to meat and carcass and female traits for MP. Recent studies in dairy populations reported a QTL in BTA 6 associated with sperm abnormality traits in Brown Swiss bulls [44, 45]. Studies in Holstein bulls identified regions in BTA 1, 2, 4, 6, 7, 8, 16, 23 and 26 associated with progressive and total motility [46]. However, none of these regions overlapped with the QTL reported in our studied population. The dissimilarities in QTL reported could be due to genetic differences between beef and dairy cattle at a genome-wide level [47].

Significant QTL mapping to the X chromosome for SC, PNS and sperm abnormalities highlights its importance in male fertility and spermatogenesis. The X chromosome is a candidate region for species divergence genes which are highly expressed in the testis of mice and humans [48]. Sexual antagonism and sex-chromosome meiotic drive have been suggested as a possible reason for the large number of genes associated with spermatogenesis found in the X chromosome [8].

### Overlapping regions across traits

Due to the vast number of genes detected for some traits, we have included the list of genes in each associated region for each trait in Additional file 4. To facilitate further use of our findings, we have included a list of all genes across associated regions in Additional file 5. A list of genes across associated regions that map at least four traits is shown in Table 5.

**Table 5:** List of candidate genes within regions associated with four out of seven traits

CHR^A^	Start-End^B^	Genes^C^	Traits^D^	N SNP < 5 × 10^− 8 E^
5	42,406,844–42,650,763	CPNE8 (1), PTPRR (1)	SC,Sheath,PNS,PD	1597
5	42,779,371–42,807,448	PTPRR (1)	SC,Sheath,PNS,PD	201
5	42,904,927–42,913,128	PTPRB (1)	SC,Sheath,PNS,PD	41
5	42,981,369–42,990,679	PTPRB (1)	SC,Sheath,PNS,PD	65
5	43,756,064–43,776,581	BEST3 (1)	SC,Sheath,PNS,PD	125
5	46,125,382–46,266,879	DYRK2 (1)	SC,Sheath,PNS,PD	681
5	46,332,642–46,628,009	CAND1 (1), ENSBTAG00000053087 (2)	SC,Sheath,PNS,PD	1295
5	47,382,308–47,645,887	GRIP1 (1), HELB (1), ENSBTAG00000053419 (1), IRAK3 (1), ENSBTAG00000052954 (1), TMBIM4 (1)	SC,Sheath,PNS,PD	1699
5	47,786,054–47,944,488	HMGA2 (1), bta-mir-763 (3)	SC,Sheath,PNS,PD	432
5	48,240,075–48,336,776	MSRB3 (1)	SC,Sheath,PNS,PD	403
5	48,438,416–48,438,418	MSRB3 (1)	SC,Sheath,PNS,MP	1

^A^Chromosome Number, ^B^Start to End location of region, ^C^Genes within regions with corresponding biotypes: (1) Protein coding, (2) lncRNA, (3) miRNA, ^D^Intersecting traits for each region. ^E^Number of SNPs that meet the significance threshold

Across traits, we observed that BTA 5 is an important region for male fertility in bulls. Regions in BTA 5 that have overlapping results point to SNP and genes associated with five out of the seven studied traits: SC, Sheath, PNS, PD and MP (Table 5). 16 candidate genes were identified within these significant regions as associated with at least four traits. Next, we reviewed the literature to discuss how the known function of these genes could be related to SC, Sheath, or sperm morphology traits.

Three candidate genes (DYRK2, CAND1, and GRIP1) listed in Table 5 have known biological roles linking them with spermatogenesis. Spermatogenesis is likely to underpin most bull fertility traits, so these genes warrant further discussion. The DYRK family of kinases displayed high expression in the testis and was suggested to play a role in the later stages of spermatogenesis [49]. The CAND1 protein is highly expressed in the brain and testis in humans and has been reported to be highly expressed in spermatozoa of fertile men [29, 50]. In mice, GRIP1 is necessary for the adhesion of Sertoli cells to germ cells and plays an important role in efficient spermatogenesis [51]. Mice without GRIP1 appeared to suffer from impaired fertility due to abnormalities in the testis [51]. However, little is known about the role of GRIP1 in bull fertility, although its gene and protein expression in different stages of the oestrous cycle have been covered previously [52]. Perhaps these genes are similarly involved with spermatogenesis in bulls. However, further research is required to ascertain their effects on bovine spermatogenesis and testicular function.

The remaining candidate genes from Table 5, do not have a known function that directly links them to spermatogenesis. However, they are ubiquitously expressed in reproductive tissues. The CPNE (copines) gene group of membrane-bound proteins have multiple functions in membrane transport, signal transduction and cancer [53]. CPNE8 is a gene expressed ubiquitously in the prostate, testis, heart, and brain tissues [53, 54]. It was previously suggested that CPNE8 might be an important gene for prostate regulation and development [54]. The PTPRR gene may have a tumour-suppressive function in prostate cancer, and prostate cancer samples often contain lower levels of PTPRR compared to regular tissue samples [55, 56]. In addition, the PTPRB gene was expressed in porcine and equine spermatozoa and found mainly in the plasma membrane of sperm heads, acrosome, and tail [57]. The expression of PTPRB mainly in the tail of spermatozoa, suggests its involvement in sperm motility regulation [57]. Previous literature has highlighted the different functions of tyrosine phosphorylation in spermatozoa, which are crucial for successful fertilisation [58–60]. The expression of the BEST3 gene in the form of bestrophin 3 is ubiquitous in human muscle but found in low levels in the bone marrow, testis and retina [61]. At the same time, BEST3 plays a role in regulating cell proliferation and apoptosis, both of which are important features in mammalian spermatogenesis [62–65]. Most of these genes appear to be involved in cancer literature, which is consistent with reproductive physiology that often involves cell proliferation [66].

Notably, three genes within BTA 5 regions (Table 5) play an important role in tropical adaptation, which is expected in this cattle population. Between 47.3 Mb and 47.9 Mb is a common region in BTA 5 that contains several genes, including HELB, which is suggested to influence tropical cattle adaptation, which helps cattle cope with harsh temperatures and high intensity of ultraviolet light [67]. IRAK3 is suggested to be involved in intramuscular fat disposition and systemic inflammation regulation, HMGA2 regulates body size. The region containing HMGA2 has been previously associated with navel length in Nellore cattle and has also been reported to regulate body size [68, 69]. A copy number variant (CNV) in the HMGA2 gene has been proposed to be a functional variant associated with naval length [68]. This CNV is within a detected QTL and may play a role in sheath score, SC, PNS, and PD in the studied population. We conducted a preliminary analysis to observe the same region (5:47,840,005-47846215, reference genome ARS UCD 1.2) and explored whether this CNV segregates in our population. Using 138 whole genome sequenced cattle, that were part of the reference panel for the SNP imputation, we observed in 79 of them an increased coverage depth which likely indicates the presence of a CNV. Future studies are required to confirm whether this region of increased coverage depth is due to a CNV segregating in our population and whether this CNV is the same as previously described. Additional efforts should be made to impute this CNV for the entire multibreed population and verify it’s contribution to these traits. Regions in BTA5 have been consistently reported in previous studies. BTA 5 is evidently harbouring important regions for fertility traits and production traits. Dissecting the genes and mutations implicated in fertility as opposed to heat tolerance or growth could further inform selective breeding.

### Variant effect prediction (VEP): candidate genes

For the most significant variant in each trait (Table 3), VEP did not reveal any variants that will have a moderate or high functional impact on a protein. Instead, most variants were labelled as modifiers which either have effects that are difficult to predict or have little evidence of protein impact.

**Fig. 5:**
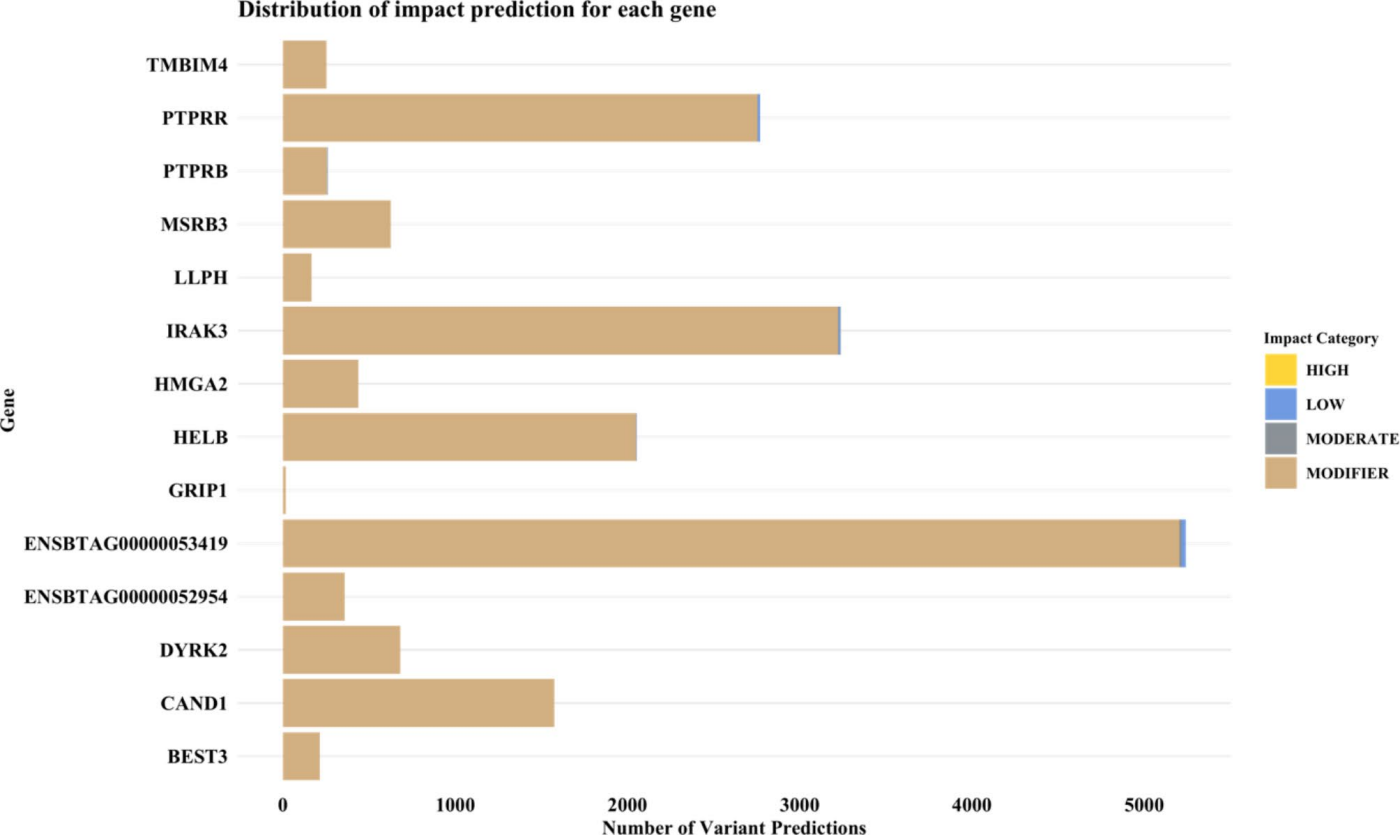
Bar plot showing the distribution of predicted impact for each gene

When VEP was expanded to include SNPs within candidate regions listed in Table 4, similar results were observed with significant variants categorised as modifiers (Fig. 5). This is logical as most traits examined in this study are complex. As such, the effects of these variants segregating in various loci across the genome have little effect on the protein or phenotype [70]. However, we observed one variant of high functional impact located in IRAK3, which results in a premature stop codon (Table 6). While this SNP may not have an equivalent quantitative effect on the trait compared to the peak SNP, it could still have a high functional impact which should be considered. As mentioned previously, IRAK3 plays a role in immune suppression. A rodent study reported a negative relationship between IRAK3 and TNF-α expression and suggested that IRAK3 is associated with immune suppression during cases of sepsis [71]. IRAK3 may also be a factor produced by Sertoli cells that causes inflammatory effector T-cells to develop regulatory functions which reduce the number of available T-cells [72]. A recent review highlighted that Sertoli cells aid in creating and maintaining an environment that shields germ cells from autoimmune destruction [73]. This is due to the presentation of antigens on the surface of end-stage germ cells, which are detected as foreign, and can lead to autoimmune destruction resulting in suboptimal fertility or sterility [73, 74]. Perhaps, a variant of high impact on IRAK3 may affect the protein’s ability to regulate autoimmune destruction efficiently, leading to decreased fertility. However, further downstream work is required to verify this speculation.

**Table 6:** Variants that were prioritised using the Variant Effect Predictor (Ensembl)

Rsid^A^	Chr:BP^B^	Consequence^C^	Gene^D^	Trait^E^
rs381450099	2:131913815	stop gained	SH2D5	SC
rs516958669	5:23029308	splice donor variant,non coding transcript variant	NUDT4	Sheath
rs209438028	5:25896459	splice acceptor variant,non coding transcript variant	SMUG1	Sheath
rs382669161	5:27216516	stop gained	KRT77	Sheath
rs136259011	5:27555357	start lost	KRT89	Sheath
rs715902417	5:28482148	start lost	BIN2	Sheath
rs516753252	5:29011232	stop gained	ENSBTAG00000038893	Sheath
rs135081036	5:31435644	stop gained	OR8S15	Sheath
rs520423926	5:34992638	stop gained	ENSBTAG00000026249	Sheath
rs465638922	5:43113646	splice donor variant	ENSBTAG00000054094	Sheath
rs524081599	5:43642979	splice acceptor variant,non coding transcript variant	MYRFL	Sheath
rs380705670	5:44348662	stop gained,splice region variant	LYSB	Sheath
rs479267746	5:47594939	stop gained	IRAK3	SC, Sheath, PNS, PD
rs209263815	5:55987506	splice donor variant	ARHGAP9	SC, Sheath
rs210582075	5:60955156	splice donor variant	CFAP54	Sheath
rs209628246	5:75712558	start lost	CYTH4	Sheath
rs439285466	X:76,501,215	splice donor variant	RLIM	SC

^A^Reference SNP cluster ID, ^B^Chromosome and Base Pair, ^C^Predicted Protein Consequence, ^D^Gene name, ^E^Trait associated

### Variants prioritized with the variant effect predictor

We identified 17 high-impact variants, predicted with VEP, as shown in Table 6. High-impact variants are predicted to have a disruptive effect on a protein, which may have a potential downstream impact on the associated phenotypes [31]. Pairwise LD calculation between high-impact variants and the top variants for their respective QTL are available in Additional file 6 (Tables S7 to S10). Among these high-impact variants, 15 variants were in BTA 5, and the remaining two were found in BTA 2 and the X chromosome. All variants were associated with either SC, Sheath, PNS or PD. Seven high-impact variants were in LD with the top variants for their respective QTL with an R^2^ ranging from 0.41 to 0.97. The high-impact variant rs479267746 lies within the coding region of a gene (IRAK3) which has been previously associated with fertility. The expression of IRAK3 by Sertoli cells, which play an important role in spermatogenesis, has been discussed in detail in the previous section. The high-impact variant rs439285466 lies within the protein-coding region of a gene called RLIM. Although RLIM has not been associated to bull fertility or bull production traits, it has been previously associated with the regulation of cell proliferation which is fundamental process for spermatogenesis [75]. Considering the LD with top QTL variants for SC and other bull traits, together with the VEP results and the known function of IRAK3 and RLIM, we would prioritize the 2 high-impact variants in these genes for future work. These variants should be further tested for their impact on bull fertility.

The remaining 10 high-impact variants, while not in LD (R^2^ < 0.4) with the top variants of the corresponding QTL, were significantly associated with either SC or Sheath themselves. Some of the high impact variants identified in this study, lie within known genes (NUDT4, SMUG1, KRT77, BIN2, ARHGAP9, and CFAP54) previously not connected with bull traits or male fertility [75–82]. We proposed these 17 variants be further investigated in subsequent analysis to ascertain variant effects in other populations.

## Conclusion

This study highlights the importance of BTA5 for bull fertility and production traits and demonstrates the need to include the X chromosome in genomic analyses. We also highlighted candidate genes of relevance across several traits, which should be further investigated in ascertaining gene effects on spermatogenesis and fertility. Finally, we identified several high-impact variants for SC and Sheath, which required further validation in future work.

## Electronic supplementary material

Below is the link to the electronic supplementary material.

Supplementary Material 1

Supplementary Material 2

Supplementary Material 3

Supplementary Material 4

Supplementary Material 5

Supplementary Material 6

## Data Availability

The raw data on which the conclusions of the paper rely are available from the CSIRO https://www.csiro.au/) under a Data Use Agreement. Summary statistics for every tested SNP are available as supplementary files (Additional files 1 to 5). The datasets can be accessed in ScienceDB using the following link: https://www.scidb.cn/s/7Jbi2e. Addtionally, the unique links for each additional file has been listed in the supporting information section below. Original phenotype data can be obtained from respective producers under circumstances where a data-sharing agreement has been reached, this can be arranged through the corresponding author.
