# Supplementary material for: Sequence level genome-wide associations for bull production and fertility traits in tropically adapted bulls
Source: BMC Genomics. 2023 Jun 29;24:365. doi: 10.1186/s12864-023-09475-2 (PMC10308662; doi:10.1186/s12864-023-09475-2)
Supplement: Supplementary file 3 — Supplementary Material 3 [file 12864_2023_9475_MOESM3_ESM.docx]

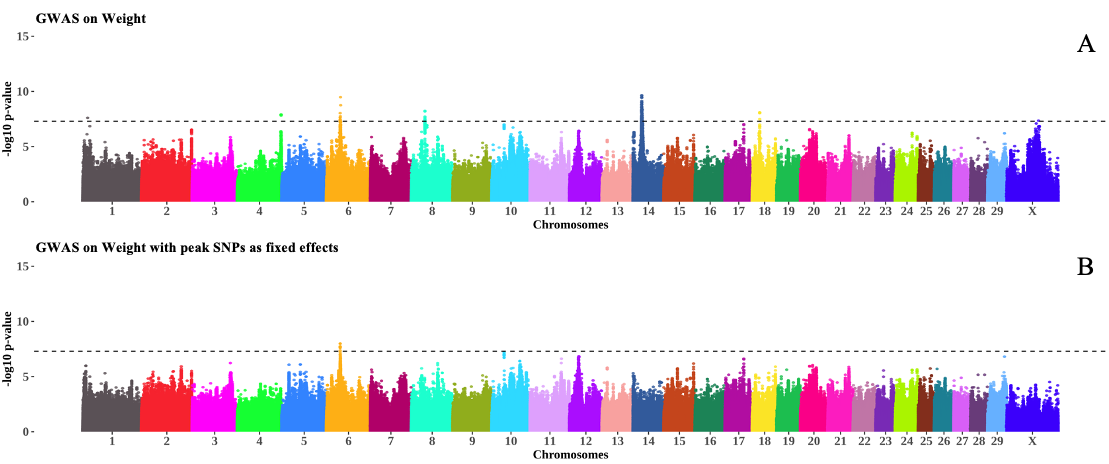


Figure S1. The Manhattan plot in A shows associations for Weight in GWAS LOCO, whereas the Manhattan plot in B shows associations for Weight after fitting the most significant SNP in each chromosome as a fixed effect. The inverse log p – values for each SNP are plotted along the y-axis for each chromosome on the x-axis. The dotted line represents the genome-wide significance threshold of 5 x 10^-8^.


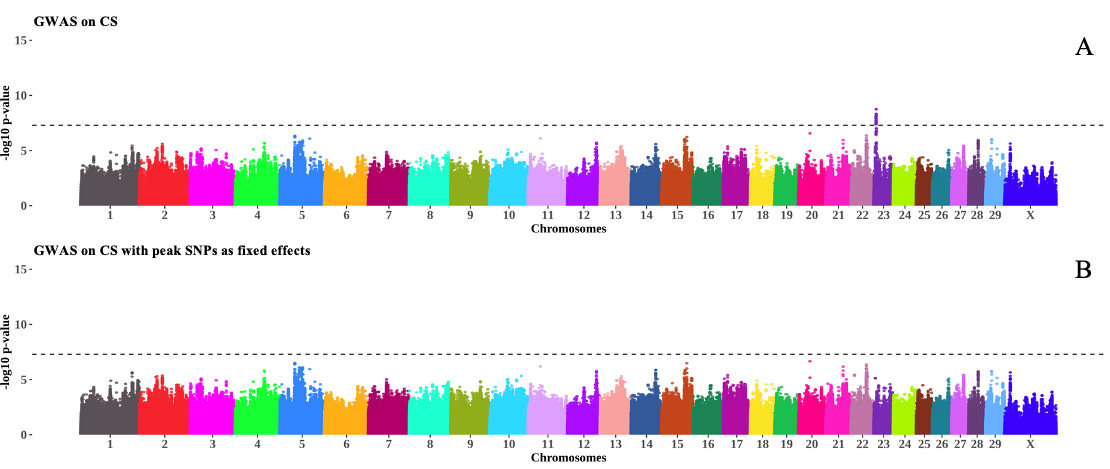


Figure S2. The Manhattan plot in A shows associations for CS in GWAS LOCO, whereas the Manhattan plot in B shows associations for CS after fitting the most significant SNP in each chromosome as a fixed effect. The inverse log p – values for each SNP are plotted along the y-axis for each chromosome on the x-axis. The dotted line represents the genome-wide significance threshold of 5 x 10^-8^.


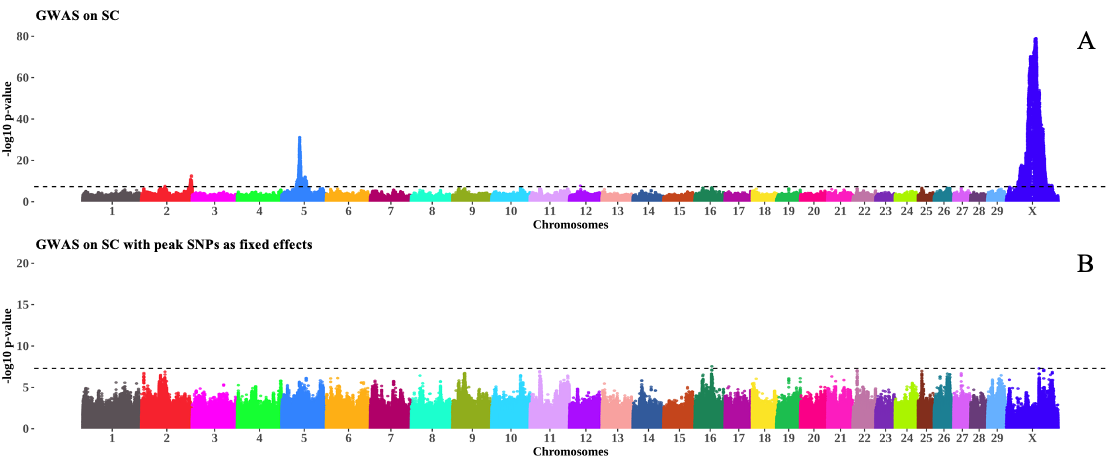


Figure S3. The Manhattan plot in A shows associations for SC in GWAS LOCO, whereas the Manhattan plot in B shows associations for SC after fitting the most significant SNP in each chromosome as a fixed effect. The inverse log p – values for each SNP are plotted along the y-axis for each chromosome on the x-axis. The dotted line represents the genome-wide significance threshold of 5 x 10^-8^.


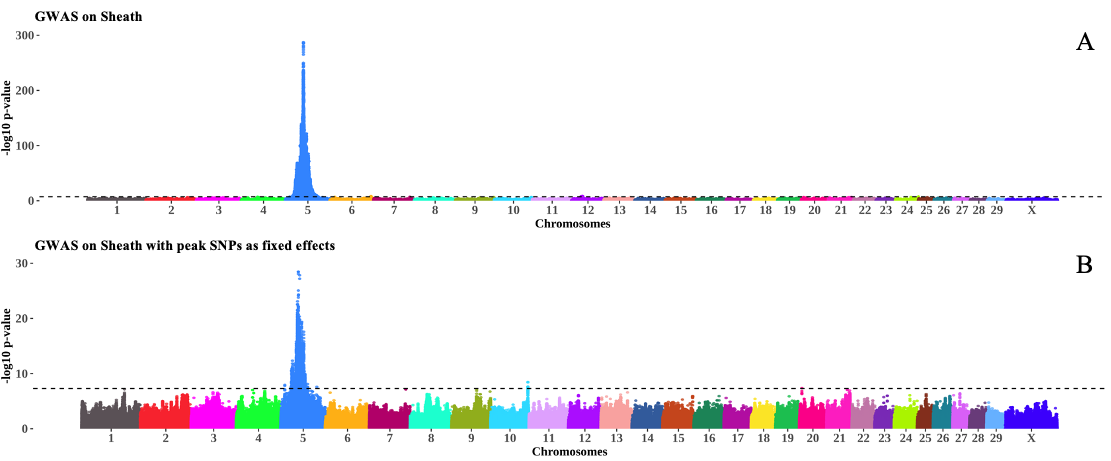


Figure S4. The Manhattan plot in A shows associations for Sheath in GWAS LOCO, whereas the Manhattan plot in B shows associations for Sheath after fitting the most significant SNP in each chromosome as a fixed effect. The inverse log p – values for each SNP are plotted along the y-axis for each chromosome on the x-axis. The dotted line represents the genome-wide significance threshold of 5 x 10^-8^.


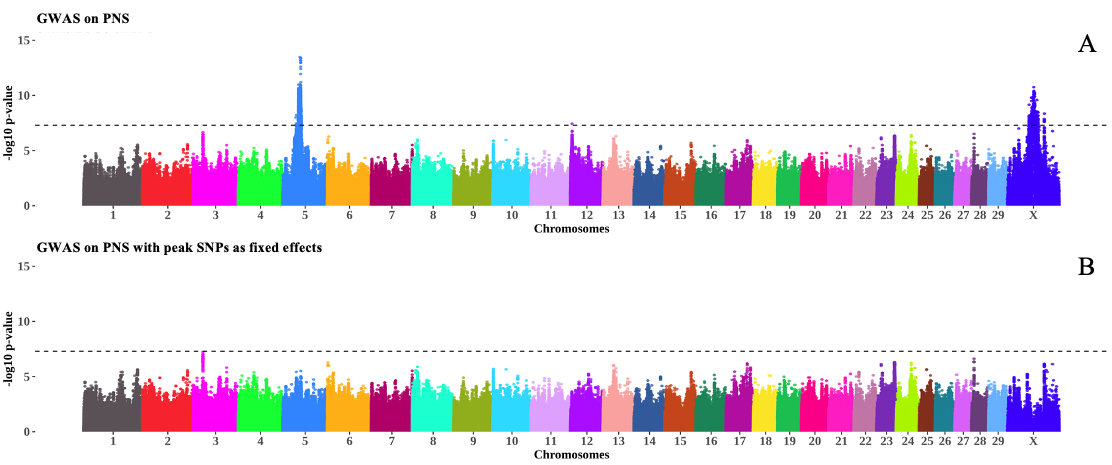


Figure S5. The Manhattan plot in A shows associations for PNS in GWAS LOCO, whereas the Manhattan plot in B shows associations for PNS after fitting the most significant SNP in each chromosome as a fixed effect. The inverse log p – values for each SNP are plotted along the y-axis for each chromosome on the x-axis. The dotted line represents the genome-wide significance threshold of 5 x 10^-8^.


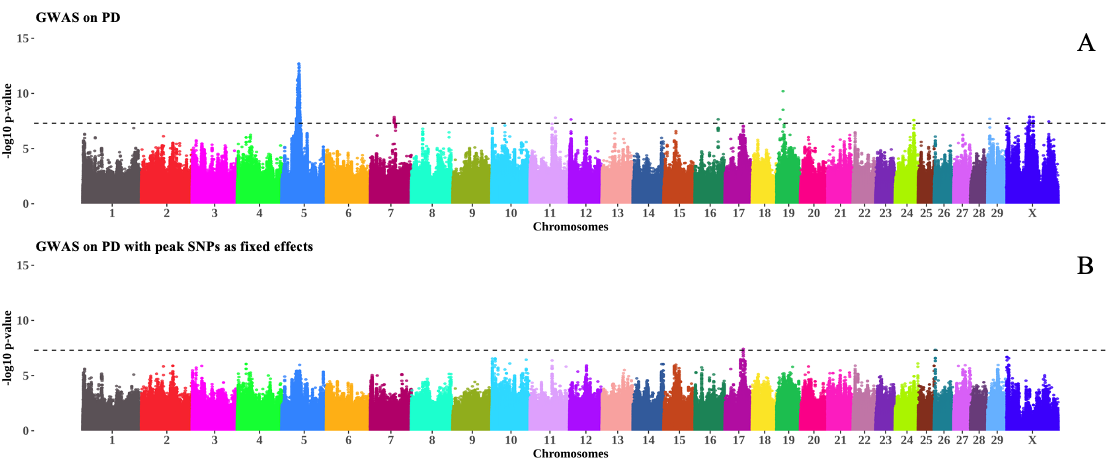


Figure S6. The Manhattan plot in A shows associations for PD in GWAS LOCO, whereas the Manhattan plot in B shows associations for PD after fitting the most significant SNP in each chromosome as a fixed effect. The inverse log p – values for each SNP are plotted along the y-axis for each chromosome on the x-axis. The dotted line represents the genome-wide significance threshold of 5 x 10^-8^.


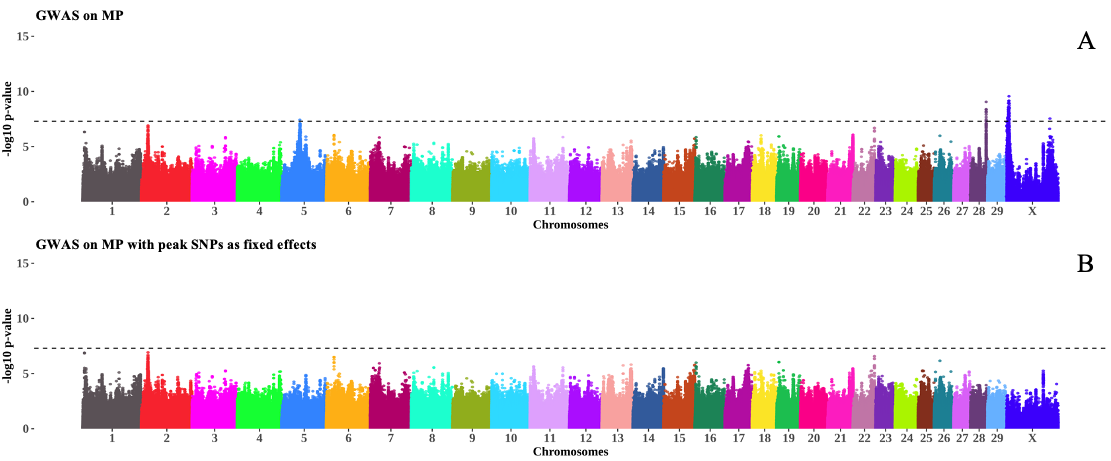


Figure S7. The Manhattan plot in A shows associations for MP in GWAS LOCO, whereas the Manhattan plot in B shows associations for MP after fitting the most significant SNP in each chromosome as a fixed effect. The inverse log p – values for each SNP are plotted along the y-axis for each chromosome on the x-axis. The dotted line represents the genome-wide significance threshold of 5 x 10^-8^.
