# Supplementary material for: Sequence level genome-wide associations for bull production and fertility traits in tropically adapted bulls
Source: BMC Genomics. 2023 Jun 29;24:365. doi: 10.1186/s12864-023-09475-2 (PMC10308662; doi:10.1186/s12864-023-09475-2)
Supplement: Supplementary file 4 — Supplementary Material 4 [file 12864_2023_9475_MOESM4_ESM.docx]

| Breed^A^ | Animals  assayed  50K SNP^B^ | Reference HD  700K SNP^C^ | Reference Sequence  25M SNP^D^ |
| --- | --- | --- | --- |
| Afrikander |  |  | 5 |
| Angus |  | 195 | 50 |
| Angus Red |  |  | 30 |
| Beefmaster |  |  | 16 |
| Belmont Tropical Composite | 660 | 130 |  |
| Bonsmara |  | 32 |  |
| Boran |  | 24 | 21 |
| Brahman | 1051 | 863 | 200 |
| Brangus |  |  | 5 |
| Charolais |  |  | 50 |
| Composite |  | 12 |  |
| Droughtmaster | 760 | 345 | 37 |
| Gir |  |  | 7 |
| Hereford |  |  | 50 |
| Limousin |  |  | 50 |
| Murray Grey |  |  | 2 |
| Nelore |  |  | 12 |
| Santa Gertrudis | 1288 | 467 | 28 |
| Senepol |  |  | 12 |
| Shaiwal |  |  | 2 |
| Shorthorn |  |  | 33 |
| Tropical Composite | 1819 | 351 | 56 |
| Tuli |  | 33 | 2 |
| Ultra-black | 844 |  |  |
| Total | **6422** | **2,452** | **668** |

Table S1. The number of animals genotype at 50K and number animals used as reference panel for imputation to High Density (HD) and subsequently to Sequence Level.

^A^Bull breed. ^B^Number of animals assayed with the 50K SNP chip. ^C^Number of animals for the reference population assayed with the 700K SNP chip. ^D^Number of animals for the reference population assayed at sequence level.

Table S2. Descriptive statistics for Brahman breed.

|  | N^A^ | Mean^B^ | SD^C^ | Min^D^ | Max^E^ |
| --- | --- | --- | --- | --- | --- |
| Weight | 1051 | 359.05 | 42.95 | 221 | 558 |
| CS | 1051 | 3.09 | 0.37 | 2 | 3.5 |
| SC | 1051 | 27.93 | 2.81 | 20.5 | 40.5 |
| Sheath | 1051 | 5.89 | 1.12 | 3 | 9 |
| PNS | 1051 | 51.58 | 30.24 | 0 | 98 |
| PD | 1051 | 24.50 | 27.16 | 0 | 91 |
| MP | 1051 | 13.40 | 11.25 | 0 | 83 |

^A^Number of records available for each trait. ^B^Mean of each trait. ^C^Standard deviation each trait. ^D^Minimum value each trait. ^E^Maximum value each trait.

Table S3. Descriptive statistics for Tropical Composite breed.

|  | N^A^ | Mean^B^ | SD^C^ | Min^D^ | Max^E^ |
| --- | --- | --- | --- | --- | --- |
| Weight | 1819 | 328.50 | 58.67 | 162 | 580 |
| CS | 1819 | 2.81 | 0.44 | 2 | 3.5 |
| SC | 1819 | 28.90 | 2.90 | 21 | 38.5 |
| Sheath | 1819 | 3.13 | 1.77 | 1 | 9 |
| PNS | 1819 | 57.63 | 27.24 | 0 | 96 |
| PD | 1819 | 14.52 | 19.79 | 0 | 96 |
| MP | 1819 | 14.35 | 12.47 | 0 | 82 |

^A^Number of records available for each trait. ^B^Mean of each trait. ^C^Standard deviation each trait. ^D^Minimum value each trait. ^E^Maximum value each trait.

Table S4. Descriptive statistics for Santa Gertrudis breed.

|  | N^A^ | Mean^B^ | SD^C^ | Min^D^ | Max^E^ |
| --- | --- | --- | --- | --- | --- |
| Weight | 1285 | 506.06 | 71.77 | 264 | 810 |
| CS | 928 | 3.03 | 0.29 | 2 | 4 |
| SC | 1274 | 34.48 | 3.06 | 25 | 52.5 |
| Sheath | 1285 | 2.81 | 0.82 | 1 | 4.5 |
| PNS | 1242 | 73.58 | 20.79 | 0 | 99 |
| PD | 1242 | 6.71 | 12.08 | 0 | 94 |
| MP | 1242 | 8.06 | 8.36 | 0 | 68 |

^A^Number of records available for each trait. ^B^Mean of each trait. ^C^Standard deviation each trait. ^D^Minimum value each trait. ^E^Maximum value each trait.

Table S5. Descriptive statistics for Drought Master breed

|  | N^A^ | Mean^B^ | SD^C^ | Min^D^ | Max^E^ |
| --- | --- | --- | --- | --- | --- |
| Weight | 750 | 459.54 | 58.05 | 334 | 743 |
| CS | 617 | 3.06 | 0.30 | 2.5 | 4 |
| SC | 601 | 33.65 | 3.17 | 23.2 | 47 |
| Sheath | 760 | 3.15 | 0.68 | 1 | 4.5 |
| PNS | 710 | 63.76 | 25.99 | 0 | 99 |
| PD | 709 | 9.89 | 16.37 | 0 | 92 |
| MP | 709 | 9.25 | 9.83 | 0 | 65 |

^A^Number of records available for each trait. ^B^Mean of each trait. ^C^Standard deviation each trait. ^D^Minimum value each trait. ^E^Maximum value each trait.

Table S6. Descriptive statistics for Ultra Black

|  | N^A^ | Mean^B^ | SD^C^ | Min^D^ | Max^E^ |
| --- | --- | --- | --- | --- | --- |
| Weight | 454 | 439.17 | 65.70 | 257 | 648 |
| CS | 842 | 3.09 | 0.24 | 2 | 4 |
| SC | 837 | 33.80 | 3.39 | 22.5 | 49 |
| Sheath | 842 | 1.78 | 0.81 | 1 | 4 |
| PNS | 783 | 67.70 | 26.55 | 0 | 100 |
| PD | 781 | 10.20 | 15.95 | 0 | 89 |
| MP | 781 | 7.83 | 8.23 | 0 | 68 |

^A^Number of records available for each trait. ^B^Mean of each trait. ^C^Standard deviation each trait. ^D^Minimum value each trait. ^E^Maximum value each trait.

Table S7. Linkage disequilibrium R^2^ between the high impact variants and the top variants associated with sheath score.

| **RSID**^A^ | **Gene**^B^ | **CHR**^C^ | **Start**^D^ | **End**^E^ | **TOP-RSID**^F^ | **TOP**^G^ | **HI**^H^ | **R^2^**^I^ |
| --- | --- | --- | --- | --- | --- | --- | --- | --- |
| rs516958669 | NUDT4 | 5 | 22980300 | 23473101 | rs381963741 | 4.25E-13 | 1.98E-08 | 0.32 |
| rs209438028 | SMUG1 | 5 | 23539577 | 38667395 | rs459004128 | 5.15E-69 | 6.93E-23 | 0.41 |
| rs382669161 | KRT77 | 5 | 23539577 | 38667395 | rs459004128 | 5.15E-69 | 3.16E-25 | 0.33 |
| rs136259011 | KRT89 | 5 | 23539577 | 38667395 | rs459004128 | 5.15E-69 | 4.81E-14 | 0.22 |
| rs715902417 | BIN2 | 5 | 23539577 | 38667395 | rs459004128 | 5.15E-69 | 1.42E-14 | 0.30 |
| rs516753252 | ENSBTAG00000038893 | 5 | 23539577 | 38667395 | rs459004128 | 5.15E-69 | 2.70E-19 | 0.18 |
| rs135081036 | OR8S15 | 5 | 23539577 | 38667395 | rs459004128 | 5.15E-69 | 5.47E-16 | 0.32 |
| rs520423926 | ENSBTAG00000026249 | 5 | 23539577 | 38667395 | rs459004128 | 5.15E-69 | 5.96E-09 | 0.03 |
| rs465638922 | ENSBTAG00000054094 | 5 | 41706627 | 44179090 | rs439264766 | 5.65E-140 | 2.72E-19 | 0.12 |
| rs524081599 | MYRFL | 5 | 41706627 | 44179090 | rs439264766 | 5.65E-140 | 2.00E-13 | 0.13 |
| rs380705670 | LYSB | 5 | 44264399 | 57689597 | rs132782818 | 8.44E-288 | 6.36E-09 | 0.00 |
| rs380705670 | LYSB | 5 | 44264399 | 57689597 | rs522682579 | 8.44E-288 | 6.36E-09 | 0.00 |
| rs380705670 | LYSB | 5 | 44264399 | 57689597 | rs137124586 | 8.44E-288 | 6.36E-09 | 0.00 |
| rs479267746 | IRAK3 | 5 | 44264399 | 57689597 | rs132782818 | 8.44E-288 | 2.47E-233 | 0.75 |
| rs479267746 | IRAK3 | 5 | 44264399 | 57689597 | rs522682579 | 8.44E-288 | 2.47E-233 | 0.75 |
| rs479267746 | IRAK3 | 5 | 44264399 | 57689597 | rs137124586 | 8.44E-288 | 2.47E-233 | 0.75 |
| rs209263815 | ARHGAP9 | 5 | 44264399 | 57689597 | rs132782818 | 8.44E-288 | 1.28E-94 | 0.46 |
| rs209263815 | ARHGAP9 | 5 | 44264399 | 57689597 | rs522682579 | 8.44E-288 | 1.28E-94 | 0.46 |
| rs209263815 | ARHGAP9 | 5 | 44264399 | 57689597 | rs137124586 | 8.44E-288 | 1.28E-94 | 0.46 |
| rs210582075 | CFAP54 | 5 | 59801206 | 66615726 | rs482771913 | 7.30E-86 | 2.89E-53 | 0.59 |
| rs210582075 | CFAP54 | 5 | 59801206 | 66615726 | rs461696950 | 7.30E-86 | 2.89E-53 | 0.59 |
| rs210582075 | CFAP54 | 5 | 59801206 | 66615726 | rs443457124 | 7.30E-86 | 2.89E-53 | 0.59 |
| rs209628246 | CYTH4 | 5 | 75565447 | 75716072 | rs385049596 | 1.02E-12 | 3.32E-10 | 0.60 |
| rs209628246 | CYTH4 | 5 | 75565447 | 75716072 | rs379949076 | 1.02E-12 | 3.32E-10 | 0.60 |

^A^Reference SNP cluster ID. ^B^Corresponding gene. ^C^Chromosome number. ^D^QTL window start coordinates. ^E^QTL window end coordinates. ^F^Reference SNP cluster ID for top variant in QTL window. ^G^Top variant in QTL window P-value for Sheath. ^H^High impact variant P-value for Sheath. ^I^Linkage Disequilibrium R^2^.

Table S8. Linkage disequilibrium R^2^ between the high impact variants and the top variants associated with SC.

| RSID^A^ | Gene^B^ | CHR^C^ | Start^D^ | End^E^ | TOP-RSID^F^ | TOP^G^ | HI^H^ | R^2I^ |
| --- | --- | --- | --- | --- | --- | --- | --- | --- |
| rs381450099 | ENSBTAG00000050449 | 2 | 131774486 | 131986530 | rs381498576 | 1.83E-09 | 4.50E-08 | 0.87 |
| rs479267746 | IRAK3 | 5 | 46771866 | 49487196 | rs133417252 | 6.18E-32 | 1.86E-26 | 0.75 |
| rs209263815 | ARHGAP9 | 5 | 55472745 | 56266503 | rs208372185 | 3.18E-11 | 2.58E-09 | 0.82 |
| rs439285466 | RLIM | X | 74094167 | 76761401 | rs136929687 | 1.44E-75 | 6.06E-73 | 0.97 |

^A^Reference SNP cluster ID. ^B^Corresponding gene. ^C^Chromosome number. ^D^QTL window start coordinates. ^E^QTL window end coordinates. ^F^Reference SNP cluster ID for top variant in QTL window. ^G^Top variant in QTL window P-value for SC. ^H^High impact variant P-value for SC. ^I^Linkage Disequilibrium R^2^.

Table S9. Linkage disequilibrium R^2^ between the high impact variant and the top variants associated with PNS.

| RSID^A^ | Gene^B^ | CHR^C^ | Start^D^ | End^E^ | TOP-RSID^F^ | TOP^G^ | HI^H^ | R^2I^ |
| --- | --- | --- | --- | --- | --- | --- | --- | --- |
| rs479267746 | IRAK3 | 5 | 47316888 | 47944488 | rs132782818 | 4.11E-14 | 7.74E-11 | 0.75 |
| rs479267746 | IRAK3 | 5 | 47316888 | 47944488 | rs522682579 | 4.11E-14 | 7.74E-11 | 0.75 |
| rs479267746 | IRAK3 | 5 | 47316888 | 47944488 | rs137124586 | 4.11E-14 | 7.74E-11 | 0.75 |

^A^Reference SNP cluster ID. ^B^Corresponding gene. ^C^Chromosome number. ^D^QTL window start coordinates. ^E^QTL window end coordinates. ^F^Reference SNP cluster ID for top variant in QTL window. ^G^Top variant in QTL window P-value for PNS. ^H^High impact variant P-value for PNS. ^I^Linkage Disequilibrium R^2^.

Table S10. Linkage disequilibrium R^2^ between the high impact variant and the top variants associated with PD.

| **RSID**^A^ | **Gene**^B^ | **CHR**^C^ | **Start**^D^ | **End**^E^ | **TOP-RSID**^F^ | **TOP**^G^ | **HI**^H^ | **R^2^**^I^ |
| --- | --- | --- | --- | --- | --- | --- | --- | --- |
| rs479267746 | IRAK3 | 5 | 47382308 | 47645887 | rs439010603 | 7.57E-09 | 1.70E-08 | 0.95 |

^A^Reference SNP cluster ID. ^B^Corresponding gene. ^C^Chromosome number. ^D^QTL window start coordinates. ^E^QTL window end coordinates. ^F^Reference SNP cluster ID for top variant in QTL window. ^G^Top variant in QTL window P-value for PD. ^H^High impact variant P-value for PD. ^I^Linkage Disequilibrium R^2^.
