# Supplementary material for: Sequence level genome-wide associations for bull production and fertility traits in tropically adapted bulls
Source: BMC Genomics. 2023 Jun 29;24:365. doi: 10.1186/s12864-023-09475-2 (PMC10308662; doi:10.1186/s12864-023-09475-2)
Supplement: Supplementary file 6 — Supplementary Material 6 [file 12864_2023_9475_MOESM6_ESM.docx]

# Supporting Information

File Name: Additional file 1

File extension: additional_file_1.txt

File Link:

<https://download.scidb.cn/download?fileId=48872c19060950b98124a949e890cc29&dataSetType=personal&username=weiliangandre.tan@uqconnect.edu.au&fileName=additional_file_1.txt>

Title: Genome-wide association studies summary statistics for all traits

Genome-wide association studies summary statistics for ~13 mb for each of the seven traits.

File Name: Additional file 2

File extension: additional_file_2.txt

File Link: <https://download.scidb.cn/download?fileId=43e0066838475f84a27cdb379b740b98&dataSetType=personal&username=weiliangandre.tan@uqconnect.edu.au&fileName=additional_file_2.txt>

Title: Genome-wide association studies summary statistics for significant SNPs

Genome-wide association studies summary statistics for only significant SNPs for each of the seven traits. This includes a SNP-wise trait count column which counts the number of trait a particular SNP is significant.

File Name: Additional file 3

File extension: additional_file_3.txt

File Link:

<https://download.scidb.cn/download?fileId=a44ba088208699636f3c03fc990db5b9&dataSetType=personal&username=weiliangandre.tan@uqconnect.edu.au&fileName=additional_file_3.docx>

Title: Manhattan plots for all traits

Genome-wide association studies Manhattan plots for all traits.

File Name: Additional file 4

File extension: additional_file_4.txt

File Link:

<https://download.scidb.cn/download?fileId=62fe34bf5d504be19e547313c69e373a&dataSetType=personal&username=weiliangandre.tan@uqconnect.edu.au&fileName=additional_file_4.txt>

Title: Gene list for significant regions in each trait.

A list of genes that overlap with significant regions for each trait.

File Name: Additional file 5

File extension: additional_file_5.txt

File Link:

<https://download.scidb.cn/download?fileId=1af67cec2976b882cd31b78a1aa15c0b&dataSetType=personal&username=weiliangandre.tan@uqconnect.edu.au&fileName=additional_file_5.txt>

Title: Gene list for significant regions across traits.

A list of genes that overlap with significant regions across several traits.

File Name: Additional file 6

File extension: additional_file_6.txt

File Link:

<https://download.scidb.cn/download?fileId=b2a2b849a3dfc082accdf69a43706af3&dataSetType=personal&username=weiliangandre.tan@uqconnect.edu.au&fileName=additional_file_6.docx>

Title: Breed-wise summary statistics and pairwise linkage disequilibrium analysis for high impact variants.

Summary statistics for each breed and pairwise linkage disequilibrium analysis results for high impact variants and their corresponding QTLs.
